# An Analysis of the Associations among Cognitive Impulsiveness, Reasoning Process, and Rational Decision Making

**DOI:** 10.3389/fpsyg.2017.02324

**Published:** 2018-01-12

**Authors:** Ana P. G. Jelihovschi, Ricardo L. Cardoso, Alexandre Linhares

**Affiliations:** Fundacao Getulio Vargas, Brazilian School of Public and Business Administration, Rio de Janeiro, Brazil

**Keywords:** impulsivity, BIS-11, reflectivity, CRT, executive functions, dual process, reasoning process, decision making

## Abstract

Impulsivity may lead to several unfortunate consequences and maladaptive behaviors for both clinical and nonclinical people. It has a key role in many forms of psychopathology. Although literature has discussed the negative impact of impulsivity, few have emphasized the relationship between cognitive impulsiveness and decision making. The aim of this study is to investigate the effects of cognitive impulsiveness on decision making and explore the strategies used by participants to solve problems. For this purpose, we apply two measures of impulsivity: the self-report Barratt Impulsiveness Scale (BIS-11) and the performance based Cognitive Reflection Test (CRT). Moreover, we evaluate participants' reasoning processes employed to answer CRT questions based on the calculation expressions, data organization, and erasures they made while answering the CRT (note that we utilized the instruments using pen and paper). These reasoning processes are related to the role of executive functions in decision making, and its relationship with impulsiveness. The sample consists of 191 adults, who were either professionals or undergraduate students from the fields of business, management, or accounting. The results show that cognitive impulsiveness may negatively affect decision making, and that those who presented the calculation to answer the CRT questions made better decisions. Moreover, there was no difference in the strategies used by impulsive vs. nonimpulsive participants during decision making. Finally, people who inhibited their immediate answers to CRT questions performed better during decision making.

## 1. Introduction

Cognitive impulsiveness may lead people to make mistakes on simple reasoning tasks. A study conducted by Frederick (2005) proved that even students from the best universities in the world, make mistakes on simple reasoning questions due to misuse of their cognitive resources. Frederick applied the Cognitive Reflection Test (CRT), a three-item task with simple reasoning problems, to measure cognitive reflection ability (reflectivity and impulsivity) on undergraduate students from well-known universities. Frederick found that Harvard students scored, on average, only 1.43, while students from Princeton University scored 1.63 (on a score ranging from 0 to 3).

These intriguing results may be explained based on what researchers call cognitive dual process (Wason and Evans, 1975; Evans, 2003; Osman, 2013). Literature has suggested that people use two types of cognitive processes: System 1 and System 2 (Stanovich and West, 2000; Kahneman and Frederick, 2002; Shafir and LeBoeuf, 2002), or Type 1 and Type 2, according to a recent study (Evans and Stanovich, 2013). While Type 1 is related to an impulsive way of thinking, Type 2 is a reflective style of decision making. Thus, even when people know how to answer specific questions and how to make good decisions, they may misjudge if they use the impulsive cognitive type.

Although it is well-known that people may provide correct or incorrect answers due to the use of different cognitive processes, the strategies that could distinguish when they are using Type 1 or Type 2 to make decisions are currently unknown. Executive functions may explain and clarify the underlying reasoning processes of different strategies performed during a rational decision making.

Executive functions are mental processes that allow us to mentally play with ideas; consider alternatives rather than being impulsive; resist temptations; solve problems; and be creative when meeting unanticipated challenges (Diamond, 2013). They act as a manager of our cognitive resources—such as planning, decision making, and flexibility—which are used to accomplish objectives, including rational and mathematical problems (Blair et al., 2008; Toll et al., 2011; Bull and Lee, 2014). Cognitive flexibility, for instance, involves being flexible enough to adjust to changed demands or priorities, to admit we are wrong, and to take advantage of unexpected situations (Diamond, 2013). Planning, in turn, plays a key role in finding satisfactory solutions for a problem (Krikorian et al., 1994), and consists of the ability to create the best way to achieve a defined goal, regarding the rank of steps and the necessary tools to accomplish it (Malloy-Diniz et al., 2014). Finally, according to Zelazo and Müller (2002), executive functions are composed of “hot” and “cool” components related to emotional and cognitive processes, respectively and, their interaction involves the ability to control impulsive behaviors.

Impulsivity has a key role in many forms of psychopathology (Verdejo-García et al., 2007; Malloy-Diniz et al., 2011), and Barratt's impulsivity model is one of the most widely applied and recognized approaches (Stanford et al., 2009) used to investigate it. According to this model, the impulsiveness personality trait is composed of three subtypes: nonplanning impulsiveness (orientation toward present and cognitive complexity), motor impulsiveness (acting on the spur of the moment), and attentional impulsiveness (lack of attention and concentration) (Patton et al., 1995). However, only two factors (inhibition control and nonplanning) have been found for adults in the Brazilian context (Vasconcelos et al., 2012; Malloy-Diniz et al., 2015). From a neurobiological perspective, the nonplanning subtype from Barratt's model is analogous to a “cognitive impulsiveness” associated with the act of making decisions (Bechara et al., 2000). According to Bechara's model, cognitive impulsiveness is related to an inability to delay gratification, which is in line with the “orientation toward the present” characteristic of Barratt's nonplanning impulsiveness subtype.

Therefore, this study investigates the effect of impulsivity on rational decision making, and explores the strategies people use to solve problems. Although Frederick contributed significantly to the literature on cognition and decision making, he and other authors using the same instrument (Toplak et al., 2011, 2014; Cueva Herrero et al., 2015; Alos-Ferrer et al., 2016; Primi et al., 2016) did not investigate whether participants could present impulsive personality traits, and did not explain the process of reasoning used to make rational decisions. Moreover, regarding psychometric measures of impulsivity, most studies that use self-report and behavioral measures have examined the relationship between impulsivity and mental disorders, such as substance abuse (Petry and Casarella, 1999; Tarter et al., 1999; McGue et al., 2001; Dougherty et al., 2009) and obesity (Fields et al., 2013), or investigated the reliability of impulsivity measures (Reynolds et al., 2006) but not the relationship between impulsivity and decision making related to logical and abstract reasoning.

Additionally, nonplanning is an important subtype of impulsivity that is difficult to evaluate, at least using self-reported measures (Barratt, 1993). Our data-collection approach provided spontaneous and ecological data to evaluate this subtype of impulsivity and to investigate participants' executive function abilities. Our data emerged from an individual and singular procedure with no explicit instructions, unlike in usual neuropsychological tasks (Heaton et al., 1993; Bechara et al., 1994; Krikorian et al., 1994). In this regard, no study has used both CRT and the Barratt Impulsiveness Scale (BIS-11) as measures of impulsivity; this study intends to fill these gaps.

Based on the discussion in the literature on cognitive dual-process, executive functions, and impulsivity, as presented in this section, we propose and test four hypotheses. Regarding the inability to delay gratification and to plan in a long-term which are characteristics of the nonplanning impulsiveness trait (Patton et al., 1995), the first hypothesis predicts that high levels of this trait negatively affect performance in rational decision making. Following the same line, for successful accomplishment of several daily activities, including the solution of rational and mathematical problems, people should clearly identify their final objective, building a plan of goals and using hierarchical organization that makes its execution feasible (Malloy-Diniz et al., 2014). Thus, the second hypothesis states that high levels of the nonplanning impulsiveness trait negatively affect manipulation of apparent information needed to solve problems. Given that appropriate performance of executive functions has a key role in achieving objectives and making decisions (Diamond, 2013), the third hypothesis suggests that the more participants manipulate data following structured reasoning, the better their performance on rational decision making. Finally, cognitive flexibility involves being flexible enough to adjust to changed demands or priorities, to admit we are wrong, and to take advantage of unexpected situations (Diamond, 2013). Hence, our fourth hypothesis predicts that people who initially provide an impulsive answer during decision making, but later rethink and change it, perform satisfactorily when solving problems—i.e., cognitive flexibility positively affects rational decision making.

## 2. Materials and methods

### 2.1. Participants

The sample was comprised of 191 participants who were professionals (74.3%) or undergraduate students (25.6%) from the fields of business, accounting, or management. It included 44.3% women, and the participants' mean age was 33.9 years (SD = 10.24). In total, 191 participants answered the survey, but seven participants did not reveal their monthly income, 11 did not answer all CRT questions, and one left one BIS-11 question unanswered. Thus, due to these missing values, the final sample for analysis was between 183 and 180 for the hypotheses tests^1^.

Participants were volunteers recruited from a well-known Brazilian entertainment company; from an executive education program in the public sector at Getulio Vargas Foundation (FGV); and from the accountancy undergraduate program at the State University of Rio de Janeiro. The latter two institutions are prestigious universities in Brazil; hence, we assume that all participants were able to read, interpret questions, and perform the four basic math operations (add, subtract, multiply, and divide). Inclusion criteria were aged above 18 years, and higher education completed or underway (participants who did not meet these criteria were excluded from the study). The study was approved by the Human Subjects Review Committee of FGV-EBAPE (Cod: 18032016-1710).

### 2.2. Instruments

Impulsivity was measured based on two validated instruments:

- BIS-11, translated version (Malloy-Diniz et al., 2010): This is a self-report Likert scale from 1 to 4 (1 = rarely/never; 4 = almost always/always) consisting of 30 items that evaluate the behavior construct and personality trait of impulsivity. This scale measures the three subtypes of impulsivity (nonplanning impulsiveness, attentional impulsiveness, motor impulsiveness) and total impulsiveness, which is the sum of the subtypes. Nevertheless, for the Brazilian context, a two-factor division (inhibition control and nonplanning impulsiveness), besides the total score, was adopted (Malloy-Diniz et al., 2010, 2015).
- CRT (Frederick, 2005): This is a performance-based task comprising the following three reasoning questions: (1) A bat and a ball cost $1.10 in total. The bat costs $1.00 more than the ball. How much does the ball cost? (2) If it takes 5 machines 5 min to make 5 widgets, how long would it take 100 machines to make 100 widgets? (3) In a lake, there is a patch of lily pads. Every day, the patch doubles in size. If it takes 48 days for the patch to cover the entire lake, how long would it take for the patch to cover half of the lake? The scores range from 0 (no correct answer) to 3 (all answers correct), and measures one type of cognitive ability: the tendency to override a premature response that is usually incorrect, and to engage in reflective reasoning, which usually leads to correct answers. Translation into Portuguese was carried out by the researchers. The first question in the CRT was adapted to the Brazilian price level reality and culture, since baseball is not popular in the country. Thus, we replaced the bat and ball with candy (*bala*) and bubble gum (*chiclete*).

### 2.3. Procedures

Participants completed the survey in a single session lasting 30 min maximum. The media corporation workers answered the survey during an event organized by their employer, while students completed it in their classrooms, after they had returned from a break.

Surveys were completed individually, with participants answering the CRT, BIS-11, and demographics questions after signing a consent form. The CRT questionnaire had a blank space for participants to use for their calculations if necessary. However, in order to avoid influencing participants on their decisions as to whether to use this blank space, nothing was said about the possibility of conducting calculations in those spaces. The researchers provided directions to respondents on how to answer the questions by themselves, without consulting external sources. When in doubt, participants were told to ask the researchers, who remained present in the classroom during the procedures for help. At the end of the survey, participants returned the sheet with their answers, along with their consent form. We stored both documents separately, and coded the participants to ensure anonymity.

With the aim of better understanding the main features of the respondents, the sample was divided among *impulsive* and *nonimpulsive* groups, following the normative parameters proposed by Malloy-Diniz et al. (2015). Participants at the 75th percentile or above on the BIS-11 total impulsiveness variable were assigned to the *impulsive* group, while those below the 75th percentile were assigned to the *nonimpulsive* group. However, to test hypotheses we used the whole sample.

For the BIS-11 assessments, we analyzed the data using a two-factor structure, as suggested by Vasconcelos et al. (2012) and Malloy-Diniz et al. (2015) for a sample comprised of Brazilian adults. The nonplanning factor was determined based on the same 11 items from the original study (Patton et al., 1995) (i.e., 1, 7, 8, 10, 12, 13, 14, 15, 18, 27, and 29). The other factor, inhibitory control, was determined based on the remaining 19 items that were originally split between motor and attentional impulsiveness factors.

In turn, the CRT was applied for the purposes of measuring rational decision making (i.e., the CRT scores) and to investigate participants' abilities to manage their executive functions during their reasoning processes. To achieve this objective, participants were required to use pen and paper, leading to more spontaneous and ecological performance-based data.

**Figure 1:**
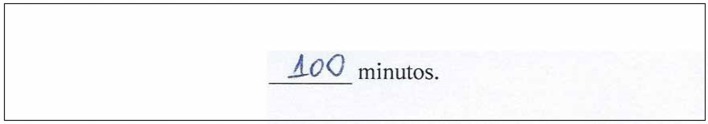
Illustration of no expression reasoning strategy. *No expression* is the no externalization or expression of calculation or reasoning.

**Figure 2:**
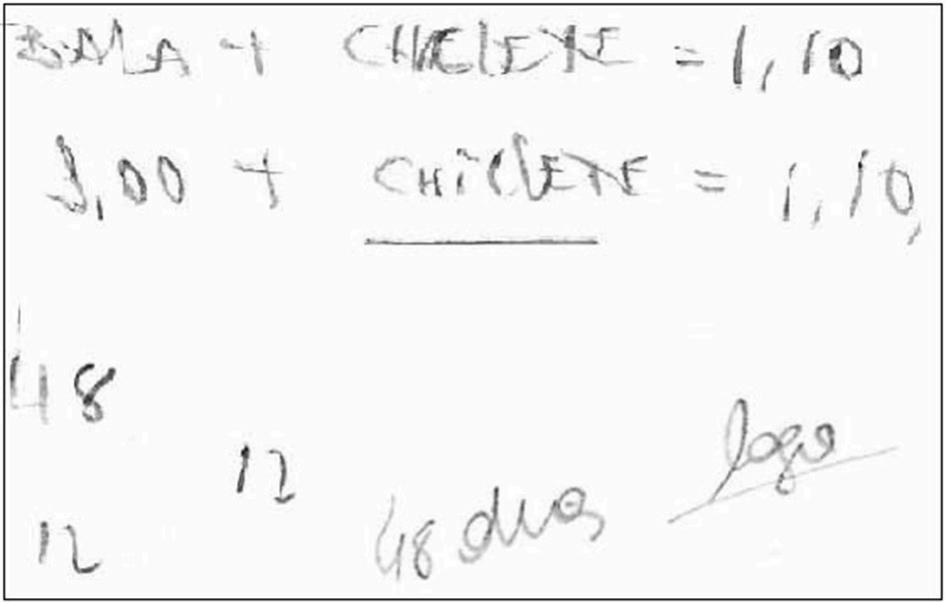
Illustration of organization reasoning strategy. *Organization* is the presentation of some organization but with no persistence regarding calculation.

**Figure 3:**
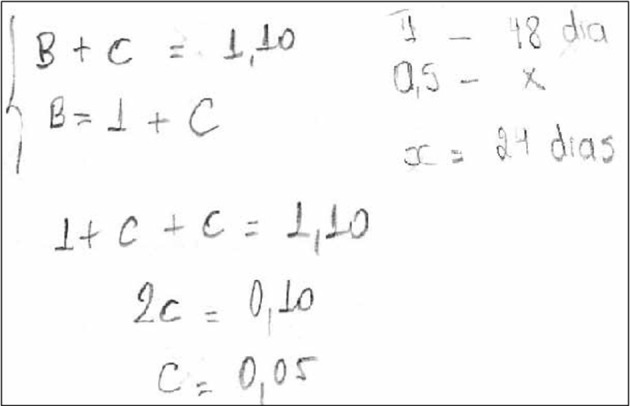
Illustration of calculation reasoning strategy. *Calculation* is the high manipulation of data, demonstrating a rationale with some structured sequences of reasoning.

**Figure 4:**
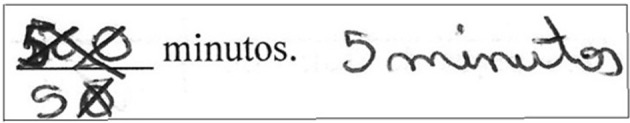
Erasure variable. *Erasure*: It seems that this participant, initially, answered impulsively, reconsidered her answer, and then changed her mind.

Thus, we were able to observe four types of reasoning process to answer the CRT: those that did not present any externalization or calculation expression or reasoning (*no expression*), as depicted in Figure 1; those that showed some data organization but with no persistence in terms of development of calculation (*organization*), as shown in Figure 2; and those with a high manipulation of data, demonstrating a rationale with some structured sequences of reasoning (*calculation*), as demonstrated in Figure 3. Interestingly, some people answered the question and then erased and changed the answer, showing calculation or not. It seems that these participants first answered impulsively, but then reconsidered their answers and changed their minds, presenting cognitive flexibility during decision making. This variable was termed as *erasure*, and is illustrated in Figure 4. As for CRT, the scores range from 0 (any organization or calculation; no organization; no calculation; or no erasure depending on the variable) to 3 (no organization or calculation; organization; calculation; or erasure to all three questions, depending on the variable).

To evaluate the strategies used by participants, we evaluated how they answered the CRT questions on their sheets. Two researchers and an assistant analyzed and coded the different types of reasoning processes of each questionnaire. To reach a consensus, each researcher evaluated each variable separately to allocate it to the observed types of reasoning processes. Following the individual analysis of each reasoning process, the researchers discussed the few divergences until consensus was reached. Data were analyzed using Stata version 14.1.

## 3. Results

Descriptive analysis was used in order to characterize the sample. Age, gender, occupation, and income are the demographic characteristics of the sample Annual income is a categorical variable ranging from US$ 7,536.23 to over US$ 48,985.50^2^.

Regarding performance on CRT, 36.9% of the subjects did not answer any question correctly; 21.9% correctly answered one question; 24.1% two questions; and 17.1% answered all three questions correctly, as depicted in Table 1.

**Table 1:** Summary statistics.

**Panel A: Quantitative variables**	**Impulsive subsample Mean (SD)**	**Nonimpulsive subsample Mean (SD)**	**Overall Mean (SD)**
Nonplanning	27.9 (3.08)	21.77 (3.63)	23.38 (4.42)
Inhibition control	43.2 (5.36)	33.14 (3.78)	35.78 (6.13)
Total impulsiveness	71.1 (5.37)	54.92 (6.03)	59.16 (9.23)
Age (years)	33.97 (9.57)	33.86 (10.49)	33.90 (10.24)
N	50	141	191
**Panel B: Qualitative variables**	**Impulsive subsample N (%)**	**Nonimpulsive subsample N (%)**	**Overall N (%)**
**CRT SCORE**			
0 (no correct answer)	20 (40.82)	49 (35.51)	69 (36.90)
1 (only one correct answer)	9 (18.37)	32 (23.19)	41 (21.93)
2 (two correct answers)	12 (24.49)	33 (23.91)	45 (24.06)
3 (all three correct answers)	8 (16.33)	24 (17.39)	32 (17.11)
**NO EXPRESSION**			
0 (organization or calculation for all three questions)	5 (10)	14 (9.93)	19 (9.95)
1 (organization or calculation for only one question)	6 (12)	12 (8.51)	18 (9.42)
2 (organization or calculation for two questions)	8 (16)	20 (14.18)	28 (14.66)
3 (no organization or calculation)	31 (62)	95 (67.38)	126 (65.97)
**ORGANIZATION**			
0 (no organization)	41 (82)	127 (90.07)	168 (87.96)
1 (organization for only one question)	7 (14)	12 (8.51)	19 (9.95)
2 (organization for two questions)	2 (4)	2 (1.42)	4 (2.09)
3 (organization for all three questions)	0	0	0
**CALCULATION**			
0 (no calculation)	33 (66)	102 (72.34)	135 (70.68)
1 (calculation for only one question)	12 (24)	19 (13.48)	31 (16.23)
2 (calculation for two questions)	3 (6)	9 (6.38)	12 (6.28)
3 (calculation for all three questions)	2 (4)	11 (7.80)	13 (6.81)
**ERASURE**			
0 (no erasure)	41 (82)	120 (85.11)	161 (84.29)
1 (erasure for only one question)	8 (16)	19 (13.48)	27 (14.14)
2 (erasure for two questions)	1 (2)	1 (0.71)	2 (1.05)
3 (erasure for all three questions)	0	1 (0.71)	1 (0.52)
**GENDER**			
0 (male)	28 (57.14)	75 (55.15)	103 (55.68)
1 (female)	21 (42.86)	61 (44.85)	82 (44.32)
**OCCUPATION**			
0 (student)	12 (24)	37 (26.24)	49 (25.65)
1 (professional)	38 (76)	104 (73.76)	142 (74.35)
**INCOME (USD PER YEAR)**			
0 (lass than US$7.536,23)	11 (22.92)	27 (19.85)	38 (20.65)
1 (US$7,536.23 to US$13,188.40)	3 (6.25)	9 (6.62)	12 (6.52)
2 (US$13,188.00 to US$18,840.57)	6 (12.50)	18 (13.24)	24 (13.04)
3 (US$18,840.57 to US$30,144.92)	7 (14.58)	27 (19.85)	34 (18.48)
4 (US$30,144.92 to US$48,985.50)	11 (22.92)	28 (20.59)	39 (21.20)
5 (above US$48,985.50)	10 (20.83)	27 (19.85)	37 (20.11)

CRT = sum of correct answers on CRT; Nonplanning = total nonplanning impulsiveness;

Inhibition control = total inhibition control impulsiveness; Organization = sum of answers using Organizations;

Calculation = sum of answers using Calculations; Erasure = sum of answers using Erasures; No expression = sum of answers using No expressions

Gender: mean = percentage of women in the respective samples

The sum of No expressions, Organization and Calculation is 3: CRT has 3

*questions and in each one participants could presented only one of the three variables*.

*Impulsive = Total impulsiveness = or > 65. Nonimpulsive = Total impulsiveness < 65*.

Table 2 presents the correlation analysis applying Pearson correlation coefficients. The investigated variables are the total of the two subtypes of impulsivity (inhibition control impulsiveness and nonplanning impulsiveness), the total impulsiveness, the sum of the *no expression, organization, calculation*, and *erasure* reasoning process variables in answering the CRT, and the CRT score (sum of correct answers in the CRT) for each respondent.

**Table 2:** Pearson cross-correlation table.

**Variables**	**1**	**2**	**3**	**4**	**5**	**6**	**7**	**8**
1-CRT								
2-Nonplanning	−0.22							
3-Inhibition control	−0.00	0.52						
4-Total impulsiveness	−0.11	0.82	0.91					
5-No expression	−0.29	0.07	−0.04	0.00				
6-Organization	0.06	0.01	0.11	0.08	−0.49			
7-Calculation	0.30	−0.08	−0.00	−0.04	−0.92	0.10		
8-Erasure	0.22	0.04	0.10	0.09	0.03	−0.08	0.00	

CRT, sum of correct answers on CRT; Nonplanning, total nonplanning impulsiveness;

Inhibition control = total inhibition control impulsiveness; Organization = sum of answers using Organizations;

*Calculation = Sum of answers using Calculations; Erasure = Sum of answers using Erasures; No expression = sum of answers using No expressions*.

The ordinary least squares (OLS) method and ordered logistic regression (Ologit) were performed to test the hypotheses based on the whole sample (i.e., the *overall* sample depicted in the last right column of Table 1). The dependent variables are interval and ordinal; thus, the results of both methods may be useful to evaluate the robustness of the findings. The OLS method presents a simple result, and its coefficient allows for direct interpretation. On the other hand, Ologit is also appropriate for the analyses since dependent variables may be ordered from 0 (highest level of impulsive trait), for the participants who did not provide any correct answer, or who did not conduct any calculations, to 3 (highest level of reflectivity), for participants who answered all three CRT questions correctly or who wrote calculations on the sheet of paper to answer all of them. Finally, both methods allow for the control of important variables that could influence outcomes of the dependent variables, such as income (Dohmen et al., 2010). Inhibition control impulsiveness was added to the model as a control variable for the nonplanning impulsiveness effects. Moreover, *organization* was used as a control variable for the effect of *calculation*^3^.

For the hypotheses tests, the results of the regression methods differed only for the first hypothesis regarding the *p*-value, as depicted in Table 3. In this regard, OLS coefficients are reported because they allow for direct interpretation. Coefficients are not standardized because the variables are on the same scale.

**Table 3:** Hypotheses tests.

	**Ordinal Least Square (OLS)**		**Ordered Logistic Regression (Ologit)**	
	**CRT**	**Calculation**	**CRT**	**Calculation**
Nonplanning	−0.05^*^	−0.01	−0.11^**^	−0.01
	(0.02)	(0.02)	(0.04)	(0.05)
Inhibition control	0.01	0.01	0.02	0.02
	(0.01)	(0.01)	(0.03)	(0.04)
Organization	0.13		0.29	
	(0.16)		(0.33)	
Calculation	0.33^***^		0.66^***^	
	(0.07)		(0.17)	
Erasure	0.44^**^		0.90^**^	
	(0.15)		(0.34)	
Gender (1 = female)	−0.38^*^	−0.11	−0.76^*^	−0.21
	(0.16)	(0.13)	(0.31)	(0.35)
Age (years)	−0.03^**^	−0.02^*^	−0.06^**^	−0.06^*^
	(0.01)	(0.01)	(0.02)	(0.03)
Occupation (1 = professional)	0.69^*^	−0.49^*^	1.37^*^	−1.70^**^
	(0.29)	(0.24)	(0.55)	(0.61)
Income (USD)	0.06	0.20^**^	0.08	0.58^**^
	(0.09)	(0.07)	(0.16)	(0.18)
N	180	183	180	183
*R*^2^	0.283	0.081		
Adjusted *R*^2^	0.245	0.050		
Pseudo *R*^2^			0.125	0.051
F	13.41	2.73		
χ^2^			60.21	16.79

*Standard errors in parentheses*.

* *p < 0.05*,

** *p < 0.01*,

*** *p < 0.001*.

*Inhibition control is a control variable for the effect of Nonplanning*.

*Organization is a control variable for the effect of Calculation*.

CRT = sum of right answers on CRT; Nonplanning = total nonplanning impulsiveness;

Inhibition control = total inhibition control impulsiveness; Organization = sum of answers using Organizations;

*Calculation = sum of answers using Calculations; Erasure = sum of answers using Erasures*.

Hypothesis 1 predicted that high levels of nonplanning impulsiveness would negatively affect performance on decision making (CRT). After entering the demographic variables and controlling for the inhibition control subtype of impulsiveness, the regressions showed that hypothesis 1 was supported (β = −0.05, *p* < 0.05). Despite the low coefficient value, this result suggests that people who are usually present-oriented and do not think carefully may make worse choices during a decision-making process compared to people with lower levels of nonplanning impulsiveness, who are more future-oriented and careful when making decisions.

Hypothesis 2 suggested that higher levels of nonplanning impulsiveness would lead people to less frequently manipulate data by performing calculations to answer the CRT questions. The results show that this hypothesis was not supported. People with higher levels of nonplanning impulsiveness would not necessarily perform fewer calculations to answer the CRT questions. That is, there is no difference between people with high levels of cognitive impulsiveness and people with low levels of cognitive impulsiveness in their strategies to answer CRT questions and make decisions.

Hypothesis 3 stated that higher levels of data manipulation, which entails a deeper development of structured reasoning and calculation to answer the CRT questions, would lead participants to perform better on CRT compared to those who did not use calculations. The hypothesis was supported (β = 0.33, *p* < 0.001). This suggests that the development of a complete thought may lead to better outcomes compared to only making notes and not expressing the reasoning and calculation behind them.

Finally, hypothesis 4 suggested that people who utilized erasures when answering the CRT questions– that is, people who gave an answer initially but then changed their mind and presented another answer– would show cognitive flexibility during rational decision making and therefore perform better in the CRT. The hypothesis was supported (β = 0.43, *p* < 0.01), revealing that those who are able to assess and reconsider immediate responses may make better decisions.

## 4. Discussion

The ability to make advantageous and rational decisions has a critical impact on several daily decisions. Impulsiveness may be one of the factors that could preclude development of this ability (Franken et al., 2008; Wittmann and Paulus, 2008). The goal of this study was to investigate the effect of impulsiveness on decision making and explore the strategies people use to solve problems. For this purpose, we applied two measures of impulsivity—BIS-11 (Patton et al., 1995) and CRT score (Frederick, 2005)—in a sample of 191 adults who were professionals or undergraduate students from the fields of business, accounting, or management.

Pen and paper were used to answer the questionnaire in order to evaluate which strategies participants used to answer the CRT questions, which were coded as *no expression, organization, calculation*, and *erasure*. Table 4 summarizes the evidence collected in this study from all tested hypotheses.

**Table 4:** Summary of results.

**Hypotheses**	**Supported?**
H1: High levels of the nonplanning impulsiveness trait negatively affect performance on rational decision making.	Yes
H2: High levels of the nonplanning impulsiveness trait negatively affect manipulation of apparent information to solve problems.	No
H3: The more participants manipulate data following structured reasoning, the better their performance on rational decision making will be.	Yes
H4: Cognitive flexibility positively affects rational decision making.	Yes

The results show some interesting findings. The outcome regarding the first hypothesis suggested that higher levels of nonplanning impulsiveness lead to worse performance on CRT. According to Klein (1999), experienced professionals present favorable performance while using the non-deliberative Type 1 cognitive style during decision making. The author suggests that this occurs because this cognitive process involves pattern matching and recognition of familiar and typical cases.

However, in unusual situations, planning has a key role in finding satisfactory solutions to a problem (Krikorian et al., 1994). Thus, in day-to-day life, nonplanning impulsiveness may appear in the form of frustration and stress, particularly in unusual situations, such as buying an apartment or car. It can lead people with higher levels of nonplanning impulsiveness to make disadvantageous choices since there may not be a logical reason for the choices made when logic is required. In turn, it may influence these people to erroneously experience feelings of incapability: they may think they are unable to make advantageous decisions, when the problem actually lies in the way they deal with the situation.

Another finding is that there was no difference between the strategies used by people with high levels of nonplanning impulsiveness and those who are nonimpulsive when it came to answering the CRT questions. Considering that the first hypothesis is supported, this second finding suggests that some people used an incorrect logical sequence of reasoning and gave wrong answers, which was confirmed by our analysis of the questionnaires. Thus, while participants with high or low levels of nonplanning impulsiveness might have followed a logical reasoning sequence while manipulating data to answer the CRT questions, this logical sequence was wrong.

An explanation for the second result may lie in the participants' difficulty in using their intuitive understanding to guide their numerical solutions as tested in a study by Ahl et al. (1992) with children. The authors evaluated the relationship between these different types of reasoning and showed how intuitive understanding influences the numerical solution. Another possibility is that in order to reduce their cognitive load, respondents used algorithmic reasoning based on superficial features of algorithms that are not related to inherent characteristics of the problem at hand, leading to insufficient development of their mathematical skills (Jonsson et al., 2014). This finding is interesting, considering that one of the sample inclusion criteria was higher education completed or underway, which implies knowledge of basic math calculations.

The third hypothesis was supported: performing calculations positively affects CRT outcome. This suggests that when people persist in doing what they had planned their outcomes are better, assuming their plan was effective. This result seems to be intuitive, but it is important to highlight the relevance of adequate employment of executive functions. Calculating requires a plan of the necessary procedures to manipulate the given information; following up on this plan; and changing the plan when relevant circumstances have changed. Thus, it represents an adequate proxy for successful implementation of important executive functions, such as planning and inhibitory control during rational decision making, which is related to higher academic success in several fields and other success over the life course (Mischel et al., 2010; Bull and Lee, 2014).

The last hypothesis suggested that people who reevaluate their answers may perform better when making decisions. Even though some participants presented incorrect answers due to impulsive reasoning, if they had changed their mind they could have achieved better results. Thus, cognitive flexibility may play an important role in achieving the best results possible when it comes to rational decision making. In this study, participants were able to rethink their choices and change their minds without facing any negative consequences; however, this may not always be possible in their daily lives. Therefore, it is important to inhibit prompted thoughts, particularly in new situations, and to evaluate options carefully and then make the best possible choice. Finally, this finding could contribute to the literature on reasoning process conflicts, which investigates the dynamic between Type 1 and Type 2 processes and the factors that lead to Type 2 engagement (Pennycook et al., 2015). The action of reevaluating the given answer may represent the process of Type 2 monitoring of the Type 1 output.

Although the present study is not focused on the variables used as controls to test the hypotheses, such as gender, age, occupation, and income, the results in this regard present interesting and significant findings. Similarly to previous studies, this study identified that gender has a significant impact on CRT scores (β = −0.40, *p* < 0.05); i.e., men scored significantly higher than did women on CRT (Frederick, 2005; Oechssler et al., 2009; Hoppe and Kusterer, 2011; Barcellos et al., 2016). Literature has suggested that women are more risk averse compared to men when it comes to uncertain decision making (Francis et al., 2015; Jain, 2015). Moreover, Frederick (2005) found that women had lower performance on CRT compared to men: women presented more impulsive answers for each question on CRT (10, 100, and 24), while men presented more diverse wrong answers to these questions (20, 500, and 1). According to Frederick, this finding suggests that men are more reflective compared to women. However, the present study evidences that gender has no impact on the decision to engage in calculation. Considering that performing calculations is an important process related to the act of reflection and making choices, this is an interesting finding that raises a question about the concepts of reflection and reflective thinking used in the literature on decision making and cognitive ability. Moreover, studies on risk behavior and gender have considered the role of social learning in the difference between men and women regard making decisions (Booth and Nolen, 2012; Booth et al., 2014), and the type of test used to evaluate risk preferences (Filippin and Crosetto, 2016), rather than inherent gender traits.

With respect to age, the results show that older participants had poorer performance compared to younger respondents during decision making (β = −0.03, *p* < 0.01), and calculated less frequently to answer the CRT (β = −0.02, *p* < 0.05). In addition, professionals performed fewer calculations compared to students to answer the CRT (β = −0.49, *p* < 0.05), but did not differ on their CRT scores. Lastly, people with higher incomes performed more calculations compared to their lower-income peers (β = 0.20, *p* < 0.01), but income did not have impact on rational decision making.

The main contributions of this study include a methodological advancement in literature on decision making and impulsiveness. Unlike extant studies on decision making that have emphasized only people's performance, this study complements such evidence by adding data about intrinsic characteristics, reasoning process, and performance. It also contributes to the literature on impulsivity by presenting evidence using BIS-11, based on the second-order sub-scale outcomes, unlike studies that have presented evidence based only on the total score.

An additional contribution of this study is its measurement of reasoning process based on the strategies participants used to answer the CRT questions. It provides an initial idea for the development of a tool to measure nonplanning impulsiveness, which is an important subtype of impulsivity that is difficult to evaluate, at least using self-report measures (Barratt, 1993). Even though there are several tests to measure motor impulsiveness, there are few tools to measure nonplanning impulsiveness (Malloy-Diniz et al., 2010). Moreover, our data-collection approach provides a different perspective on the evaluation of executive functions. Such data emerged from an individual and singular procedure with no explicit instructions, unlike in usual neuropsychological tasks (Heaton et al., 1993; Bechara et al., 1994; Krikorian et al., 1994). Thus, it provided more spontaneous and ecological data. Finally, to our knowledge, this is the first study to have used, simultaneously, BIS-11 and CRT as measures of cognitive impulsiveness.

This study presents a few limitations, such as the fact that while some people did not performed calculations or other reasoning expressions by hand on paper, they may have done so mentally or using other resources, such as the surface of the table or their own palm. Another limitation is that the sample is restricted to specific fields of study and professionals who have similar specialties.

**Figure 5:**
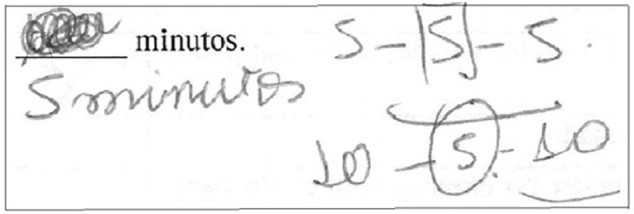
Erasure and calculation.

Moreover, we were unable to observe the relationship between *calculation* and *erasure* when participants presented both on their CRT answers. That is, we are not aware of the sequence performed by the participants, in terms of whether they first answered with no calculation, then rethought, then performed the calculation, and finally changed their answer (*erasure*); or first performed the calculations and answered the questions, thought about it and performed more calculations, and then changed their answer (*erasure*), as shown in Figure 5. Finally, data collection was not performed in a standardized way, since some of the data was collected during a company event and some was collected in classrooms.

Future research could investigate the performance of reasoning tasks related to neuropsychological tools that evaluate inhibitory control, decision making, attention, and nonverbal fluency. Furthermore, it would be fruitful to evaluate people's cognitive effort and awareness of self–impulsiveness more deeply. Thus, it would be possible to investigate the issue of compensatory behavior and whether participants are presenting higher cognitive effort compared to the presence or absence of calculations in their answers.

Regarding sample analyses, replication of this study with different participants would be valuable. That is, it would be interesting to investigate the performance on CRT, cognitive processes, and impulsiveness traits with students from different fields and professionals with other specialties. Another suggestion is to measure the time a participant takes to answer each CRT question in order to investigate the relationship between time and impulsive decision making, and to collect information about the processes of reasoning for more detailed analyses. Osman (2013) pointed out that few studies have measured response time in tasks that assess dual processes. Time measurement could contribute to the reliability of findings regarding the differences between Type 1 and Type 2 processes of reasoning. Moreover, assessment of emotions and somatic markers could provide important insights about reasoning processes, and investigation of cognitive overload effects on CRT performances may also contribute to research in this field.

Another interesting future research possibility is the exploration of recent findings about a Type 3, together with Type 1 and Type 2 reasoning processes (Noël et al., 2013). Noël and colleagues suggested that a third neural system is responsible for craving sensations and, consequently, for addictions such as gambling and drug addiction. This third system is an insula-dependent system that is responsible for the reception of interoceptive signals and their translation into feeling states, presenting significant influence in decision making and impulse control related to risk, reward, and uncertainty. Thus, a study that tests and explores this theory using CRT, BIS-11, and other useful tools to measure the association between insula, impulse control, and decision making would represent a significant contribution to both literature and the field. Finally, physiological measures such as brain activation using functional magnetic resonance imaging or electroencephalogram could be used to assess the coherence between neurophysiological activation, behavior, and feelings.

## Author contributions

AJ: Contributed substantially to the conception and design of the work; conducted the survey and statistical analysis; and wrote the manuscript. RC: Contributed substantially to the conception and design of the work; conducted the recruitment of participants; contributed to the statistical analysis and wrote the manuscript. AL: Revised the manuscript critically for important intellectual content, and had an organizational role.

### Conflict of interest statement

The authors declare that the research was conducted in the absence of any commercial or financial relationships that could be construed as a potential conflict of interest.

## Footnotes

^1^Table 1 in the Supplementary Material presents the hypotheses tests using variables with no missing values, which shows that the results do not change in this case.

^2^Participants were asked their monthly income in the Brazilian currency (Real, BRL), which was converted into U.S. dollars at the average rate for the period of data collection (i.e., USD 1 = BRL 3.45) and then multiplied by 13 (i.e., 12 months plus the thirteenth salary).

^3^Table 2 in the Supplementary Material presents the analyzes of hypotheses tests without demographic variables in any of the models.
