# Supplementary material for: An Analysis of the Associations among Cognitive Impulsiveness, Reasoning Process, and Rational Decision Making
Source: Front Psychol. 2018 Jan 12;8:2324. doi: 10.3389/fpsyg.2017.02324 (PMC5770582; doi:10.3389/fpsyg.2017.02324)
Supplement: Supplementary file 1 [file Table1.PDF]

frontiers

Supplementary Material:
An analysis of the associations among cognitive impulsiveness, reasoning process, and rational decision making

Ana Paula G. Jelihovschi*, Ricardo Lopes Cardoso, Alexandre Linhares
*Correspondence:
Author Name: Ana Paula Gomes Jelihovschi
anapgj@gmail.com

1 SUPPLEMENTARY TABLES

Table 1: Hypotheses Tests with No Missing Value

	**Ordinal Least Square (OLS)**		**Ordered Logistic Regression (Ologit)**	
	CRTs	Calculation	CRT	Calculation
Nonplanning	-0.05*	-0.01	-0.11**	-0.01
	(0.02)	(0.02)	(0.04)	(0.05)
Inhibition control	0.01	0.01	0.02	0.02
	(0.01)	(0.01)	(0.03)	(0.04)
Organization	0.13		0.29	
	(0.16)		(0.33)	
Calculation	0.33***		0.66***	
	(0.07)		(0.17)	
Erasure	0.44**		0.90**	
	(0.15)		(0.34)	
N	180	180	180	180
R^2	0.283	0.078		
Adjusted R^2	0.245	0.046		
Pseudo R^2			0.125	0.048
F	13.41	2.53		
χ^2			60.21	15.74

Standard errors in parentheses
* $p<0.05$, ** $p<0.01$, *** $p<0.001$
Demographic variables (income, age, gender, occupation) were added in all models as controls.
Inhibition control is a control variable for the effect of Nonplanning.
Organization is a control variable for the effect of Calculation.
CRT = Sum of correct answers on CRT; Nonplanning = total nonplanning impulsiveness;
Inhibition control = total inhibition control impulsiveness; Organization = sum of answers using *Organizations*;
Calculation = sum of answers using *Calculations*; Erasure = sum of answers using *Erasures*

Table 2: Hypotheses Tests with No Demographic Variables as Control

	Ordinal Least Sqaure (OLS)		Ordered Logistic Regrssion (Ologit)	
	CRT	Calculation	CRT	Calculation
Nonplanning	-0.07***	-0.02	-0.13***	-0.03
	(0.02)	(0.02)	(0.04)	(0.04)
Inhibition control	0.03	0.01	0.05	0.02
	(0.02)	(0.01)	(0.03)	(0.03)
Organization	0.11		0.25	
	(0.18)		(0.32)	
Calculation	0.34***		0.61***	
	(0.07)		(0.15)	
Erasure	0.54***		1.04***	
	(0.15)		(0.32)	
N	187	191	187	191
R^2	0.193	0.009		
Adjusted R^2	0.170	-0.002		
Pseudo R^2			0.081	0.001
F	12.16	0.85 χ^2		
41.15	0.65			

Standard errors in parentheses

* $p<0.05$, ** $p<0.01$, *** $p<0.001$

Inhibition control is a control variable for the effect of Nonplanning.

Organization is a control variable for the effect of Calculation.

CRT = sum of correct answers on CRT; Nonplanning = total nonplanning impulsiveness;

Inhibition control = total inhibition control impulsiveness; Organization = sum of answers using *Organizations*;

Calculation = sum of answers using *Calculations*; Erasure = sum of answers using *Erasures*
